# A Pharmacogenomics-Based In Silico Investigation of Opioid Prescribing in Post-operative Spine Pain Management and Personalized Therapy

**DOI:** 10.1007/s10571-024-01466-5

**Published:** 2024-05-27

**Authors:** Kai-Uwe Lewandrowski, Alireza Sharafshah, John Elfar, Sergio Luis Schmidt, Kenneth Blum, Franklin Todd Wetzel

**Affiliations:** 1 Division of Personalized Pain Therapy Research & Education, Center for Advanced Spine Care of Southern Arizona, Arizona, USA; 2 Department of Orthopaedics, Fundación Universitaria Sanitas and Member of Colombian National Academy of Medicine, Bogotá, DC Colombia; 3 Department of Orthopedics, Doctor honoris causa Hospital Universitário Gaffree Guinle Universidade Federal do Estado do Rio de Janeiro, and Member of the Brazilian National Academy of Medicine, Rio de Janeiro, Brazil; 4 Cellular and Molecular Research Center, School of Medicine, Guilan University of Medical Sciences, Rasht, Iran; 5 Department of Orthopaedics and Sports Medicine, University of Arizona College of Medicine, Tucson, AZ USA; 6 Department of Neurology, Federal University of Rio de Janeiro (UNIRIO), University Hospital, Rua Mariz e Barros 750, Tijuca, Rio de Janeiro, RJ Brazil; 7 Division of Nutrigenomics, SpliceGen, Therapeutics, Inc., Austin, TX 78701 USA; 8 Department of Psychiatry, Wright State University Boonshoft School of Medicine, Dayton, OH 45435 USA; 9 Division of Addiction Research & Education, Center for Sports, Exercise, & Mental Health, Western University Health Sciences, Pomona, CA 91766 USA; 10 The Kenneth Blum Behavioral & Neurogenetic Institute, LLC., Austin, TX 78701 USA; 11 Department of Orthopaedic Surgery & Sports Medicine, Director of Musculoskeletal Services Bassett Healthcare Network 1 Atwell Road, Cooperstown, NY 13326 USA; 12 Center for Advanced Spine Care of Southern Arizona, 4787 E Camp Lowell Drive, Tucson, USA

**Keywords:** Pharmacogenomics, Opioid, Spine pain management, Variant, Drug

## Abstract

**Abstract:**

Considering the variability in individual responses to opioids and the growing concerns about opioid addiction, prescribing opioids for postoperative pain management after spine surgery presents significant challenges. Therefore, this study undertook a novel pharmacogenomics-based in silico investigation of FDA-approved opioid medications. The DrugBank database was employed to identify all FDA-approved opioids. Subsequently, the PharmGKB database was utilized to filter through all variant annotations associated with the relevant genes. In addition, the dpSNP (https://www.ncbi.nlm.nih.gov/snp/), a publicly accessible repository, was used. Additional analyses were conducted using STRING-MODEL (version 12), Cytoscape (version 3.10.1), miRTargetLink.2, and NetworkAnalyst (version 3). The study identified 125 target genes of FDA-approved opioids, encompassing 7019 variant annotations. Of these, 3088 annotations were significant and pertained to 78 genes. During variant annotation assessments (VAA), 672 variants remained after filtration. Further in-depth filtration based on variant functions yielded 302 final filtered variants across 56 genes. The Monoamine GPCRs pathway emerged as the most significant signaling pathway. Protein–protein interaction (PPI) analysis revealed a fully connected network comprising 55 genes. Gene–miRNA Interaction (GMI) analysis of these 55 candidate genes identified miR-16-5p as a pivotal miRNA in this network. Protein–Drug Interaction (PDI) assessment showed that multiple drugs, including Ibuprofen, Nicotine, Tramadol, Haloperidol, Ketamine, l-Glutamic Acid, Caffeine, Citalopram, and Naloxone, had more than one interaction. Furthermore, Protein–Chemical Interaction (PCI) analysis highlighted that ABCB1, BCL2, CYP1A2, KCNH2, PTGS2, and DRD2 were key targets of the proposed chemicals. Notably, 10 chemicals, including carbamylhydrazine, tetrahydropalmatine, Terazosin, beta-methylcholine, rubimaillin, and quinelorane, demonstrated dual interactions with the aforementioned target genes. This comprehensive review offers multiple strong, evidence-based in silico findings regarding opioid prescribing in spine pain management, introducing 55 potential genes. The insights from this report can be applied in exome analysis as a pharmacogenomics (PGx) panel for pain susceptibility, facilitating individualized opioid prescribing through genotyping of related variants. The article also points out that African Americans represent an important group that displays a high catabolism of opioids and suggest the need for a personalized therapeutic approach based on genetic information.

**Graphical Abstract:**

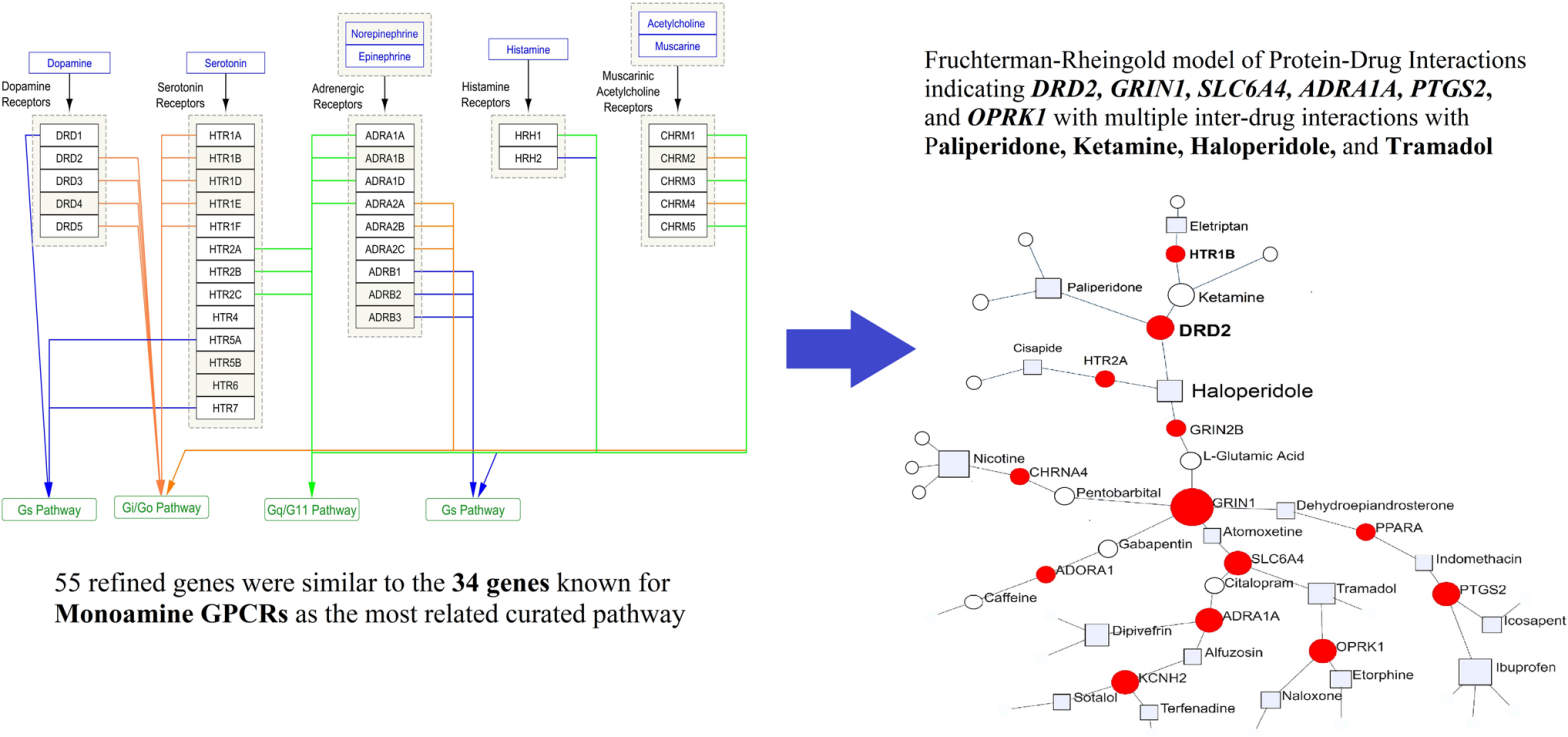

## Introduction

The United States has been grappling with a concerning rise in issues related to prescription misuse, opioid use disorder, and fatal overdoses (El Ibrahimi et al. 2023; Wang et al. 2023). Over two decades, from 1999 to 2020, the country witnessed a staggering toll of over 263,000 lives lost due to overdoses linked to prescription opioids (Aaron 2023). This distressing figure represents a nearly fivefold increase in overdose deaths involving prescription opioids from 1999 to 2020 (Böttcher et al. 2023). The root of this crisis can be traced back to the 1990s, a period when the volume of opioids being prescribed to patients started to escalate (Friedman et al. 2020). Unfortunately, as the prescription quantities of opioids surged, so did the incidents of overdoses and fatalities stemming from these medications. Despite this surge in opioid prescriptions for pain management, there has been no corresponding alteration in the amount of pain reported by the American populace (Piper et al. 2018).

The shift in medical practice has been partially ascribed to misleading information disseminated by pharmaceutical companies, who erroneously downplayed the risk of addiction associated with certain opioid pain relievers (Sarpatwari et al. 2017). This deceit had an effect since the truth regarding the highly addictive potential of these drugs recently became known (Meier 2003, 2018; Posner 2021).

By then, many of these opioids had already been misused, abused, or diverted. Concurrently, the American Pain Society introduced the notion of pain as the “fifth vital sign,” advocating for its evaluation and treatment at par with traditional vital signs such as temperature, blood pressure, respiratory rate, and heart rate (Krauss et al. 2021). This concept gained traction in 2001 with the endorsement by the Joint Commission, placing pain management practices under closer scrutiny (Scher et al. 2018).

2020 saw a grim average of 44 deaths daily due to prescription opioid overdoses, culminating in over 16,000 fatalities (Wilson et al. 2020). Almost a quarter (24%) of all deaths due to overdoses that year were related to prescription opioids; this suggests a 16% increase in prescription opioid-related morbidity between 2019 and 2020. Post-surgical pain management often marks patients' initial exposure to opioids, consequently posing a documented risk for chronic opioid dependence among opioid-naïve individuals. Alarmingly, around 70% of those using prescription opioids acquire them through diversion, frequently via legitimate prescriptions of friends and family (Krauss et al. 2021). Many surgeons, including orthopedic specialists who are ranked as the third-highest opioid prescribers, administer opioids to control postoperative pain. However, miscalculations in assessing the extent of postoperative pain can lead to excessive opioid prescriptions after surgery, inadvertently contributing to the surplus of unused pills that can be diverted (Orosz et al. 2022). The following distinct challenges are common in pain management of spine patients:

Substantial postoperative opioid needs have been associated with spine surgeries, as evidenced by recent studies encompassing various orthopedic and non-orthopedic elective procedures (Wyles et al. 2021).

Data extrapolated from the Global Burden of Disease, Injuries, and Risk Factors Study spanning 1990 to 2017 identifies low back pain as a leading cause of global disability, persistently ranking among the top five worldwide (Wu et al. 2020).

Many patients are prescribed opioids before consulting a spine surgeon, rendering them opioid-experienced, consequently heightening the risk of opioid misuse, abuse, and dependency (Cram et al. 2019).

The population of the United States is living longer, and undergoing more spine surgery. For example, between 2020 and 2040, surgical procedures for Anterior Cervical Discectomy and Fusion (ACDF) are anticipated to grow by 13.3% (from 153,288 to 173,699 procedures), while Posterior Cervical Discectomy and Fusion (PCDF) surgeries are expected to see a 19.3% increase (from 29,620 to 35,335 procedures). The most significant growth for ACDF is forecasted in the 45–54 age bracket (from 42,077 to 49,827) and the 75–84 age bracket (from 8065 to 14,862). For PCDF, the 75–84 age bracket is projected to experience the most substantial rise (from 3710 to 6836). With the demographic shift toward an older population, slight upticks are also predicted in the over-85 age group for ACDF (from 858 to 1847) and PCDF (from 730 to 1573) (Neifert et al. 2020).

Despite the identification of specific predictors that influence patterns of opioid overuse, (Dasgupta et al. 2018) such as prior opioid use, surgery type, patient age, body mass index (BMI), diagnoses of depression or anxiety, length of hospital stay, and pain scores at discharge, there is a notable absence of precise pharmacogenetic parameters in preoperative protocols. The integration of genetic factors into these protocols could be instrumental in preparing patients for postoperative pain and in determining the most effective pain management strategies. Given that opioids are among the top 30 medications with significant pharmacogenetic implications, the prospect of leveraging genetic information to guide perioperative and postoperative pain management is promising, yet its practical application remains limited (Sadhasivam and Chidambaran 2012). In this context, a patient's genetic profile related to opioid susceptibility and responsiveness could potentially replace traditional pain management methods, reducing the risks associated with opioid use and identifying more effective treatment alternatives (Manworren 2015). The most potential annotations related to the candidate genes were extracted, refined, and presented in detail (Gene, RS ID, Function, Related Drug, Genotype-phenotype associations) (Table 1).

**Table 1:** The most potential variant annotations of 55 candidate genes with structural functions

Gene	Variant	Function	Drugs	Association
ABCB1	rs2229107	Missense	Phenytoin	Allele A is associated with increased plasma drug levels of phenytoin in people with no disease as compared to genotype TT
ABCB1	rs9282564	Missense	Tacrolimus	Genotypes CC + CT is associated with decreased dose-adjusted trough concentrations of tacrolimus in people with Kidney Transplantation as compared to genotype TT
ADRA1A	rs1048101	Missense	Disulfiram	Genotypes AA + AG is associated with increased response to disulfiram in people with Cocaine-Related Disorders as compared to genotype GG
ADRA2A	rs1800035	Missense/promoter	Dexmedetomidine	Genotypes CG + GG are associated with decreased response to dexmedetomidine in women with Pain, Postoperative as compared to genotype CC
ADRB1	rs1801252	Missense/promoter	Verapamil	Genotypes AA + AG are associated with increased risk of Death when treated with verapamil in people with Coronary Artery Disease as compared to genotype GG
ADRB1	rs1801253	Missense/promoter	Carvedilol	Genotype CC is associated with increased improvement in left ventricular function (LVEF) when treated with carvedilol in people with Heart Failure as compared to genotype GG
ANKK1; DRD2	rs1800497	Missense	Bupropion; naltrexone	Genotypes AA + AG are associated with increased response to bupropion and naltrexone in people with Obesity as compared to genotype GG
BCHE	rs1799807	Missense	Succinylcholine	Allele C is associated with postanesthesia apnea when exposed to succinylcholine as compared to allele T
BCHE	rs1803274	Missense	Cocaine	Genotype TT is associated with Cocaine-Related Disorders when exposed to cocaine in as compared to genotypes CC + CT
BCL2	rs1800477	Missense/promoter	Interferons; ribavirin	Allele T is associated with increased frequency (about double) in non-responder patients compared with responder patients (HCV genotype 4) when treated with interferons and ribavirin in people with Hepatitis C, Chronic as compared to allele C
CACNA1H	rs61734410	Missense	Ethosuximide	Allele T is associated with decreased clinical benefit to ethosuximide in children with Epilepsy as compared to allele C
CES1	rs114119971	Missense	Methylphenidate	Genotype CG is associated with decreased dose of methylphenidate in children with Attention Deficit Disorder with Hyperactivity as compared to genotype GG
CFTR	rs77010898	Missense/stop-gained	Ataluren	Allele A is associated with response to ataluren in children with Cystic Fibrosis as compared to allele G
CHRNA3	rs3743075	Missense	Nicotine	Allele T is associated with decreased severity of Tobacco Use Disorder due to nicotine in men as compared to allele C
CHRNA3; CHRNA5	rs16969968	Missense	Drugs used in nicotine dependence	Allele G is associated with increased response to Drugs used in nicotine dependence in people with Tobacco Use Disorder
CHRNA4	rs1044396	Missense	Nicotine	Allele G is associated with increased risk of Tobacco Use Disorder when exposed to nicotine as compared to allele A
CYP1B1	rs1056837	Missense	Codeine; tramadol	Allele A is associated with decreased clinical benefit to codeine or tramadol in men with Pain as compared to allele G
CYP2D6	rs35742686	Frameshift	Hydrocodone; oxycodone	Allele DELT is associated with increased clinical benefit to hydrocodone or oxycodone in people with Pain as compared to allele T
CYP2D6	CYP2D6*1; CYP2D6*4; CYP2D6*5; CYP2D6*10; CYP2D6*14; CYP2D6*21; CYP2D6*36; CYP2D6*41	Missense	Tamoxifen	CYP2D6*4 + *5 + *10 + *14 + *21 + *36 + *41 are associated with increased risk of Recurrence when treated with tamoxifen in women with Breast Neoplasms as compared to CYP2D6*1/*1
CYP2D6	CYP2D6*1; CYP2D6*5; CYP2D6*17 rs28371706(T) rs16947(A)	Missense	Codeine; debrisoquine; dextromethorphan; metoprolol	CYP2D6*5/*17 is associated with decreased metabolism of codeine, debrisoquine, dextromethorphan or metoprolol in healthy individuals as compared to CYP2D6*17/*17 + *1/*1
CYP2D6	CYP2D6*1; CYP2D6*4; CYP2D6*9 (rs5030656; K281del)	Missense/deletion	Tramadol	CYP2D6*1/*4 + *1/*9 (assigned as intermediate metabolizer genotype phenotype) are associated with increased exposure to tramadol in healthy individuals as compared to CYP2D6*1/*1 (assigned as normal metabolizer genotype phenotype)
CYP3A5	rs41303343	Frameshift	Tacrolimus	Allele A is associated with decreased clearance of tacrolimus in people with Kidney Transplantation as compared to allele del
CYP3A7	rs2257401	Missense	Tacrolimus	Allele C is associated with trough concentration of tacrolimus in people with Kidney Transplantation and Transplantation as compared to allele G
DRD3	rs6280	Missense	Risperidone	Genotypes CC + CT is associated with increased response to risperidone in children with Autistic Disorder as compared to genotype TT
GABRA2	rs279858	Missense	Sevoflurane	Genotypes CC + CT are associated with decreased mean arterial pressure when exposed to sevoflurane in people with otorhinolaryngology surgery as compared to genotype TT
GP1BA	rs6065	Missense	Aspirin	Genotype CC is associated with increased risk of aspirin resistance when treated with aspirin in healthy individuals as compared to genotypes CT + TT
HTR2A	rs6314	Missense	Antidepressants	Genotypes AG + GG are associated with increased response to antidepressants in people with Depressive Disorder, Major as compared to genotype AA
HTR2C	rs6318	Missense	Escitalopram	Allele C is associated with decreased Pain when treated with escitalopram in men with neuropathic pain as compared to allele G
KCNH2	rs1137617	Missense/stop-gained	Calcium channel blockers; nitrendipine	Genotypes AA + AG are associated with increased reduction in blood pressure when treated with calcium channel blockers or nitrendipine in people with Essential hypertension as compared to genotype GG
OPRM1	rs1799971	Missense	Ethanol; naltrexone	Genotypes AG + GG are associated with increased severity of intoxication when exposed to ethanol and naltrexone in healthy individuals as compared to genotype AA
OPRM1	rs1799972	Missense	Cocaine; ethanol; nicotine	Genotype TT is associated with increased risk of Substance-Related Disorders due to cocaine, ethanol or nicotine in women as compared to genotypes CC + CT
OPRM1	rs540825	Missense	Fentanyl	Allele T is associated with increased likelihood of Vomiting due to fentanyl in women with Pain, Postoperative as compared to allele A
OPRM1	rs562859	Missense	Heroin	Allele C is associated with increased risk of Heroin Dependence due to heroin as compared to allele T
OPRM1	rs62638690	Missense	Cocaine; heroin	Allele T is associated with decreased likelihood of Cocaine-Related Disorders or Heroin Dependence due to cocaine or heroin as compared to allele G
OPRM1	rs6912029	Missense/promoter	Cotinine	Allele T is associated with increased concentrations of cotinine in people with Heroin Dependence as compared to allele G
OPRM1	rs677830	Missense/stop-gained	Acetaminophen/tramadol	Genotypes CT + TT is associated with increased likelihood of Constipation when treated with acetaminophen/tramadol in people with Pain, Postoperative as compared to genotype CC
PPARG	rs1801282	Missense	Pioglitazone	Genotype CG is associated with increased response to pioglitazone in people with Diabetes Mellitus, Type 2 as compared to genotype CC
PTGS1	rs5789	Missense	Antiinflammatory agents, non-steroids	Allele C is associated with decreased likelihood of aspirin-induced asthma when exposed to Antiinflammatory agents, non-steroids in people with Asthma as compared to allele A
SLC6A2	rs5569	Missense	Methylphenidate	Genotype GG is associated with increased response to methylphenidate in children with Attention Deficit Disorder with Hyperactivity as compared to genotypes AA + AG
TLR4	rs4986791	Missense	Prednisolone	Allele T is associated with decreased clinical benefit to prednisolone in children with Thrombocytopenia as compared to allele C
TRPV1	rs8065080	Missense	Cannabinoids	Genotype CC is associated with increased clinical benefit to cannabinoids in people with Pain as compared to genotypes CT + TT

One potentially important tool may reside in a validated Genetic Addiction Risk Severity (GARS®) test that measure risk polymorphisms across ten reward genes having heuristic value to predetermine high genetic risk for opioid dependence. It is important to consider the interaction of neurotransmitters and genes that control the release of dopamine in the Brain Reward Cascade (BRC). It is well-known that variations within the BRC, whether genetic or epigenetic, may predispose people to addictive behaviors and alter pain tolerance. The GARS, which is the first test to accurately predict vulnerability to pain, addiction, and other compulsive behaviors, is defined as Reward Deficiency Syndrome (RDS). Moreover, sensitivity to pain may reside in the mesolimbic projection system, where genetic polymorphisms are associated with a predisposition to pain vulnerability or tolerance. Blum’s laboratory statistically validated the GARS test in 74,566 case–control subjects with Alcohol Use Disorder (AUD). This analysis assessed the Hardy–Weinberg Equilibrium (HWE) of each single nucleotide polymorphism (SNP) in controls and cases. If available, the Pearson's *χ*^2^ test or Fisher's exact test was utilized for comparing the gender, genotype, and allele distributions. The statistical analyses found the OR, 95% CI for OR, and a post-risk for 8% estimation of the population's alcoholism prevalence showed a significant detection. The OR results indicated significance for COMT, OPRM1, DRD2, DRD3, DRD4, DAT1, and 5HTT at 5% (Blum et al. 2018, 2020, 2021; Blum et al. 2022a, b; Blum et al. 2022a, b; Fried et al. 2020; Moran et al. 2021; Bajaj et al. 2022; Dennen et al. 2022; Gondré-Lewis et al. 2022; Gupta et al. 2022; Vereczkei et al. 2022; Thanos et al. 2023a, b; Thanos et al. 2023a, b). Consequently, this study aimed to explore the most effective pharmacogenomic approach to managing spinal pain in postoperative patients by analyzing potential actionable genetic variants associated with FDA-approved drugs targeting specific receptors, that is, the genes themselves (Table 2).

**Table 2:** The most potential variant annotations of 55 candidate genes with regulatory functions

Gene	Function	Variant	Drugs	Association
ABCB1	Enhancer	rs12535512	Risperidone	Genotypes CC + CT are not associated with response to risperidone in people with Schizophrenia as compared to genotype TT
ABCB1	Enhancer	rs3789243	Antiepileptics	Allele G is associated with increased risk of drug resistance when treated with antiepileptics in men with Epilepsy
ADORA1	CTCF	rs16851030	Aspirin	Genotype TT is associated with increased risk of aspirin-intolerant asthma when exposed to aspirin in people with Asthma as compared to genotypes CC + CT
ADORA2A	Promoter	rs3761422	Methotrexate	Allele T is associated with increased risk of adverse events when treated with methotrexate in people with Arthritis, Rheumatoid as compared to allele C
ADRA2A	Promoter/CTCF	rs1800544	Dexmedetomidine	Genotype GG is associated with decreased dose of dexmedetomidine in people with surgery as compared to genotypes CC + CG
CALM1	Promoter	rs12885713	Cyclosporine	Allele T is associated with increased response to cyclosporine in people with Psoriasis as compared to allele C
CAT	Promoter	rs1001179	Ethanol	Allele T is associated with increased risk of Alcoholism when exposed to ethanol as compared to allele C
CHRM1	Promoter	rs2075748	Clozapine	Genotype CC is associated with decreased dose of clozapine in people with Schizophrenia as compared to genotypes CT + TT
CHRNA3	Promoter	rs7170068	Cotinine	Allele A is associated with increased concentrations of cotinine in people with Tobacco Use Disorder as compared to allele G
CHRNA4	Enhancer	rs2236196	Nicotine	Allele G is associated with increased risk of Tobacco Use Disorder when exposed to nicotine as compared to allele A
CHRNB2	Promoter	rs2072658	Nicotine	Genotype AG is associated with increased response to nicotine in people with daily smoking as compared to genotype GG
CHRNB4	Enhancer	rs10851907	Cotinine	Allele A is associated with increased concentrations of cotinine in people with Tobacco Use Disorder as compared to allele G
CNR1	Enhancer	rs806368	Cocaine	Allele C is associated with decreased risk of Cocaine-Related Disorders when exposed to cocaine as compared to allele T
CYP1A2	CTCF	rs2069526	Escitalopram	Allele G is associated with increased metabolism of escitalopram in people with Depressive Disorder, Major as compared to allele T
CYP2D6	Splicing	CYP2D6*1; CYP2D6*1xN; CYP2D6*3; CYP2D6*4; CYP2D6*6	Codeine	CYP2D6*1xN/*1 (assigned as ultrarapid metabolizer phenotype phenotype) is associated with increased metabolism of codeine in healthy individuals as compared to CYP2D6 normal metabolizer
CYP3A5	Splicing/enhancer	rs776746	Tacrolimus	Genotype CC is associated with decreased dose of tacrolimus in people with Kidney Transplantation as compared to genotypes CT + TT
DRD2	Enhancer	rs12364283	Amphetamine	Genotype AA is associated with decreased stop reaction time when treated with amphetamine in healthy individuals as compared to genotypes AG + GG
DRD2	Enhancer	rs4436578	Clozapine; olanzapine; risperidone	Genotype CC is associated with increased risk of body weight gain when treated with clozapine, olanzapine or risperidone in people with Schizophrenia
DRD2	Promoter	rs1799732	Ethanol	Genotype GG is associated with increased severity of Alcoholism due to ethanol as compared to genotypes G/del + del/del
EGF	Promoter	rs4444903	Cetuximab	Genotype AA is associated with increased survival when treated with cetuximab in people with Colorectal Neoplasms as compared to genotypes AG + GG
GABRA2	Promoter	rs11503014	Cocaine	Allele G is associated with increased severity of Cocaine-Related Disorders due to cocaine as compared to allele C
GRIN1	Promoter	rs1126442	Methamphetamine	Genotype GG is associated with increased risk of Psychotic Disorders when exposed to methamphetamine in men with methamphetamine dependence as compared to genotypes AA + AG
GRIN2B	Enhancer	rs2058878	Acamprosate	Allele A is associated with increased response to acamprosate in people with Alcoholism as compared to allele T
GRIN2B	Splicing/promoter	rs1019385	Valproic acid	Genotype AC is associated with decreased dose of valproic acid in people with Epilepsy as compared to genotype AA
HTR1B	CTCF	rs9361235	Fluoxetine	Genotypes CC + TT is associated with increased response to fluoxetine in children with Depressive Disorder, Major as compared to genotype CT
HTR1B	Promoter	rs130058	Clomipramine; liothyronine; lithium; nefazodone; venlafaxine	Allele A is associated with decreased risk of suicidal ideation when treated with clomipramine, liothyronine, lithium, nefazodone or venlafaxine in people with Depression as compared to allele T
HTR1B	Promoter/CTCF	rs11568817	Escitalopram	Genotypes AC + CC is associated with increased risk of adverse cognitive effects when treated with escitalopram in people with Anxiety Disorders as compared to genotype AA
HTR1B	Promoter/CTCF	rs6296	Citalopram	Genotype CC is associated with increased risk of agitation when treated with citalopram in children with Anxiety Disorders or Depressive Disorder, Major as compared to genotypes CG + GG
HTR2A	Enhancer	rs2770296	Bupropion	Genotype CC is associated with increased response to bupropion in people with Depressive Disorder, Major as compared to genotypes CT + TT
HTR2A	Promoter	rs6311	Citalopram; escitalopram; fluoxetine; paroxetine; sertraline	Genotype CC is associated with increased likelihood of Sexual Dysfunctions, Psychological when treated with citalopram, escitalopram, fluoxetine, paroxetine or sertraline in people with Depression as compared to genotypes CT + TT
HTR2A	Promoter	rs6313	Antidepressants	Genotype GG is associated with increased response to antidepressants in people with Depressive Disorder, Major as compared to genotypes AA + AG
HTR2C	Enhancer	rs9698290	Risperidone	Genotype TT is associated with increased response to risperidone in women with Schizophrenia as compared to genotypes CC + CT
HTR3A	Promoter/CTCF	rs1062613	Clozapine	Allele C is associated with increased response to clozapine in people with Schizophrenia as compared to allele T
HTR7	Open chromatin	rs1935349	Atorvastatin; pravastatin; simvastatin	Allele T is associated with increased risk of Myalgia unspecified when treated with atorvastatin, pravastatin or simvastatin in people with Hypercholesterolemia as compared to allele C
OPRK1	Promoter	rs1051660	Morphine	Allele A is associated with decreased dose of morphine in people with Pain as compared to allele C
OPRL1	Promoter	rs6090041	Opioids	Allele G is associated with increased risk of Opioid-Related Disorders when exposed to opioids as compared to allele A
OPRM1	CTCF/enhancer	rs2281617	Amphetamine	Genotype CC is associated with increased Euphoric and Energetic after amphetamine (10 mg) when exposed to amphetamine in healthy individuals as compared to genotypes CT + TT
OPRM1	Enhancer	rs10485058	Methadone	Genotype AA is associated with increased response to methadone in people with Opioid-Related Disorders as compared to genotypes AG + GG
OPRM1	Enhancer	rs9397685	Fentanyl	Genotype AG is associated with decreased severity of Nausea or Vomiting due to fentanyl in people with Pain, Postoperative as compared to genotype AA
PPARA	Enhancer	rs4253778	Atenolol; Beta Blocking Agents; carvedilol; metoprolol	Genotype GG is associated with decreased cardiac rehospitalization when treated with atenolol, Beta Blocking Agents, carvedilol or metoprolol in people with Acute coronary syndrome as compared to genotypes CC + CG
PPARG	Open chromatin	rs3856806	Aspirin	Genotype TT is associated with increased risk of aspirin hypersensitivity when treated with aspirin in people with Asthma as compared to genotypes CC + CT
PTGS1	Promoter	rs10306114	Aspirin	Allele G is associated with increased risk of non-response to aspirin when treated with aspirin in people with Coronary Artery Disease as compared to genotype AA
SCN2A	Splicing	rs17183814	Carbamazepine; phenobarbital; phenytoin; valproic acid	Allele A is associated with resistance to carbamazepine, phenobarbital, phenytoin or valproic acid as compared to allele G
SLC6A2	CTCF	rs2242446	3, 4-Methylenedioxymethamphetamine	Genotype TT are associated with decreased response to 3, 4-methylenedioxymethamphetamine as compared to genotypes CC + CT
SLC6A2	Enhancer	rs12708954	Atomoxetine	Allele A is associated with increased response to atomoxetine in children with Attention Deficit Disorder with Hyperactivity as compared to allele C
SLC6A4	CTCF	rs25531	Venlafaxine	Genotype TT is associated with increased reduction in HAM-A when treated with venlafaxine in people with Anxiety Disorders as compared to genotypes CC + CT
SLC6A4	Enhancer	rs2066713	Ethanol	Allele A is associated with increased risk of Alcoholism due to ethanol as compared to allele G
TACR1	Enhancer	rs735668	Opioids	Genotype AA is associated with increased risk of Opioid-Related Disorders due to opioids in women as compared to genotypes AC + CC

CTCF refers to CCCTC-binding site which is a negative regulatory for expression and enhancer is a positive regulatory binding site for transcription factors

## Methods

This study designed a bioinformatics strategy first and then reviewed the PubMed data for the keywords such as opioids, spine surgery, spine pain, pharmacogenomics, and personalized medicine. These keywords were considered the main focus of publications filtering the papers containing opioid use in spine surgery for example. To reach a precise archive of related researches, some studies were excluded from this review due to the lack of if they failed to highlight how opioids may impact spine pain management. Thus, this investigation is divided into two main categorizations; in sillico computational database analysis investigations and systematic review of the literature. The publically available and accessible dpSNP database was searched (https://www.ncbi.nlm.nih.gov/snp/) for our reference variants.

### In Silico Methods

Documenting the DrugBank results of approved drugs having a role in opioid addiction, commonly used drugs such as methadone, morphine, and amphetamine were selected. The database was search for targets associated with these drugs. Following this step, all the targets were merged and duplicated targets were omitted. The in silico analyses carried out in the current study included Variant annotation assessment (VAA) by PharmGKB (https://www.pharmgkb.org/), signaling pathways (Cytoscape ver. 3.10.1), protein–protein interactions (PPI) by STRING-MODEL (https://string-db.org/), Gene–miRNA interactions (GMI) by miRTargetLink2 (https://ccb-compute.cs.uni-saarland.de/mirtargetlink2), and Protein–drug interactions (PDI) and Protein–Chemical Interactions (PCIs) by NetworkAnalyst (https://www.networkanalyst.ca/NetworkAnalyst/). The results of all aforementioned interactions are described in the various sub-sections of the “Results” section.

## Results

### Protein–Protein Interactions (PPI)

**Fig. 1:**
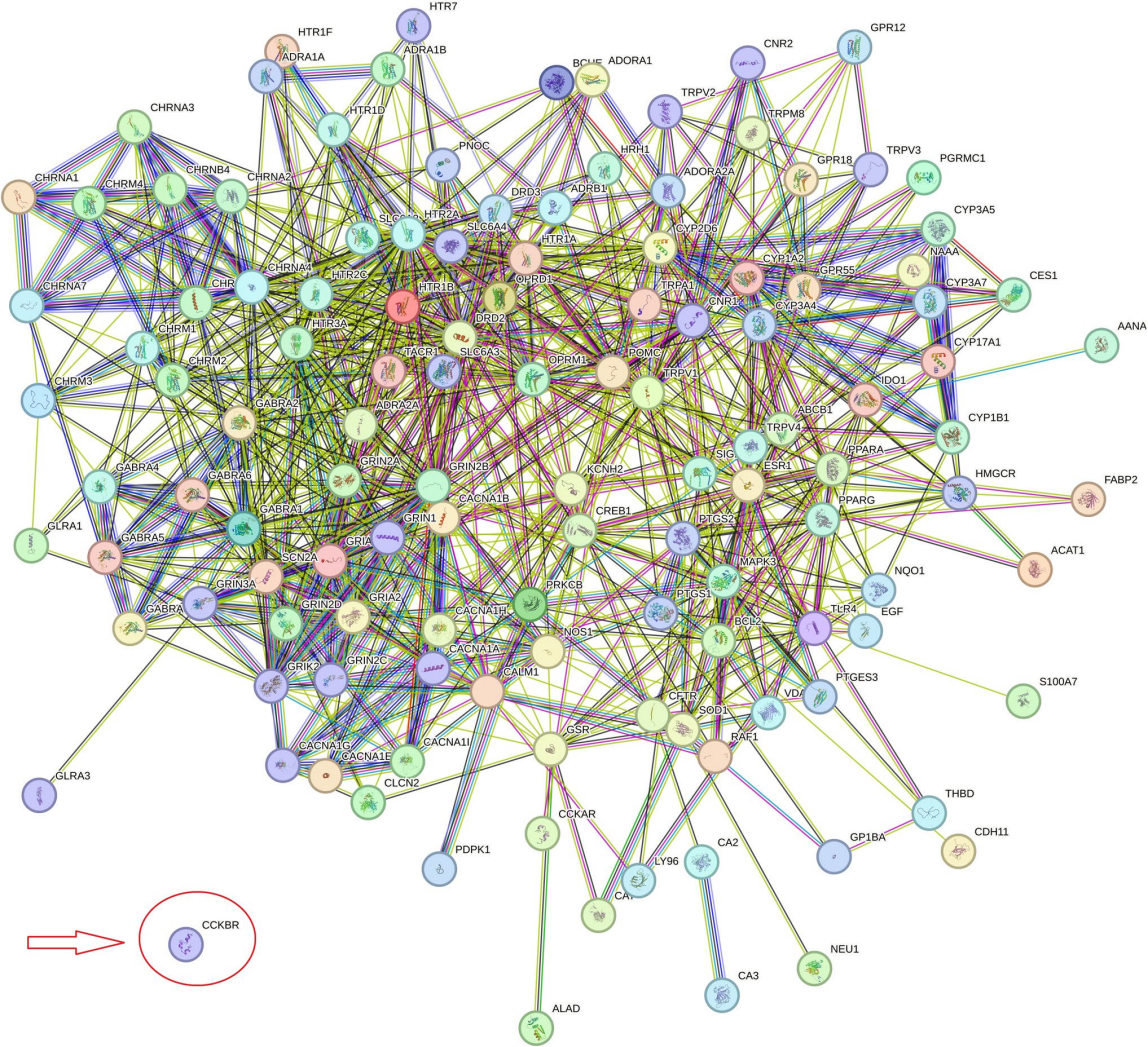
STRING-MODEL of 126 extracted targets of FDA-approved drugs from DrugBank visualized by a PPI enrichment *p*-value lower than 1.0e−16

A large network of 126 opioid targets was extracted by STRING MODEL via the DrugBank search. Almost all of the proteins were connected in this network except CCKBR. Hence, further analysis was done with the 125 remaining genes. PPI enrichment *p*-value was lower than 1.0e−16. The other molecularly-demonstrated fully-connected targets were selected for further analyses (Fig. 1).

### Variant Annotation Assessment (VAA)

Due to the importance of pharmacogenomics on spine pain management, the 125 remaining genes from PPIs, were then checked for their variant annotations. In total, 7019 variant annotations were found, among them, 3088 had a significant annotation according to the *p*-value below 0.05. Furthermore, 47 genes were discarded for having no variant annotations or not significant annotations and 78 candidate genes remained. To be more specific, the genotype associations of variant annotations were then searched for “pain” or “addiction.” Finally, after omitting the unrelated genotype associations and duplicated annotations, 78 genes and 672 variants remained. Additional deep filtrations based on the functions of variants indicated that there were 302 in total including 222 functional and 80 regulatory variants (29 Promoter, 25 Enhancer, 7 CTCF, 14 Splicing, and 5 overlapped functions variants). The intronic, intergenic, ncRNA, and synonymous variants were discarded. These 302 final filtered variants were related to the 56 genes. Another gene was eliminated as a result of the STRING MODEL described in the following, reducing the number of genes to 55.

### Signaling Pathway Assessment (SPA)

**Fig. 2:**
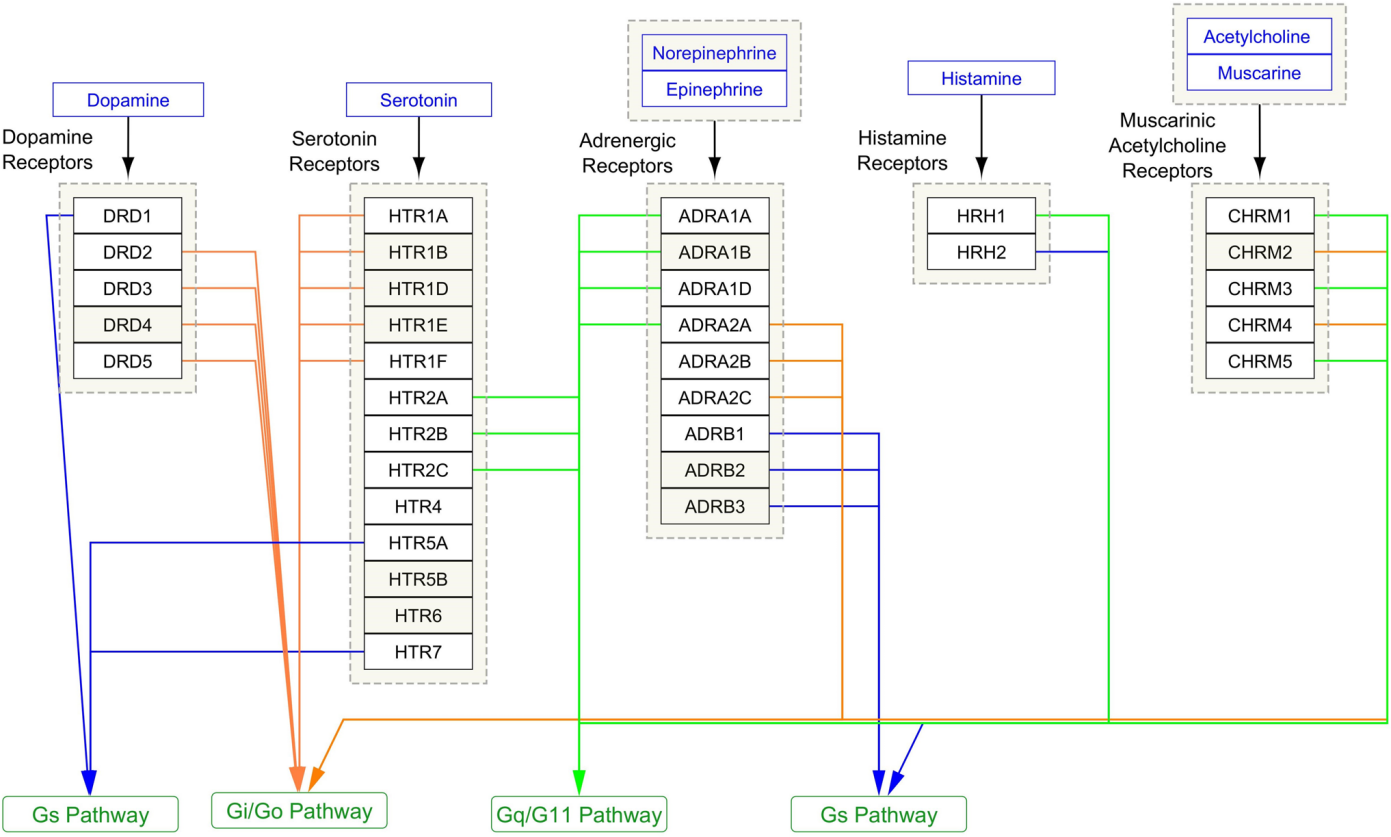
The Cytoscape output of 55 genes showing Monoamine GPCRs as the most significant pathway with a *p*-value of 3.78e−13

Cytoscape analysis was carried out to find the signaling pathways which might be helpful for checking the refined gene list (55 genes) and prioritize the genes with higher potential of being drug targets based on the pharmacogenomics approach. Results revealed that 10 genes of out of 55 refined genes were similar with the 34 gene list of Monoamine GPCRs (10 out of 34) as the most related curated pathway with the *p*-value of 3.78e−13. These 10 genes were: ADRA1A, ADRA2A, ADRB1, CHRM1, DRD2, DRD3, HTR1B, HTR2A, HTR2C, and HTR7 (Fig. 2). The second most significant pathway was based on the nicotine effect on dopaminergic neurons with a *p*-value of 1.12e−7.

### Gene–miRNA Interactions (GMIs)

**Fig. 3:**
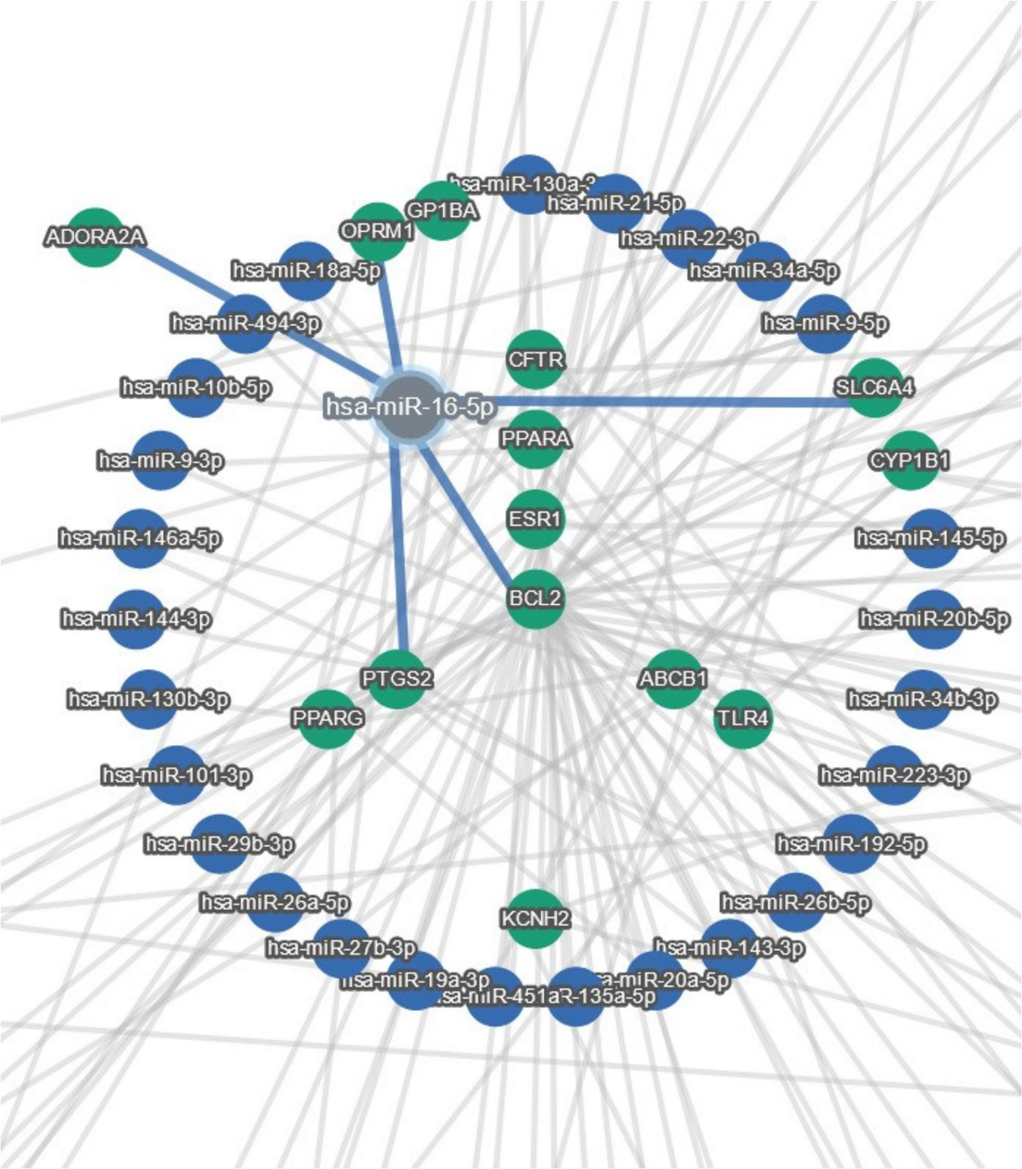
The concentric model of 55 genes adjusted with strong validated evidences illustrated by miRTargetLink2 representing the most important genes and miRNAs in the candidate opioid gene list

miRTargetLink2 was applied on the 55 refined genes and adjusted with the strong molecular evidences to reveal the most important genes according to the GMIs. In the center of the concentric model, 13 genes had the maximum shared interactions including *ABCB1, OPRM1, SLC6A4, CYP1B1, GP1BA, PPARA, TRL4, KCNH2, PPARG, PTGS2, BCL2, CFTR*, and *ESR1*. Additionally, one miRNA indicated the maximum shared relationships with the aforementioned genes in the center of the concentric model including hsa-miR-16-5p (Fig. 3).

### Protein–Drug Interactions (PDIs)

**Fig. 4:**
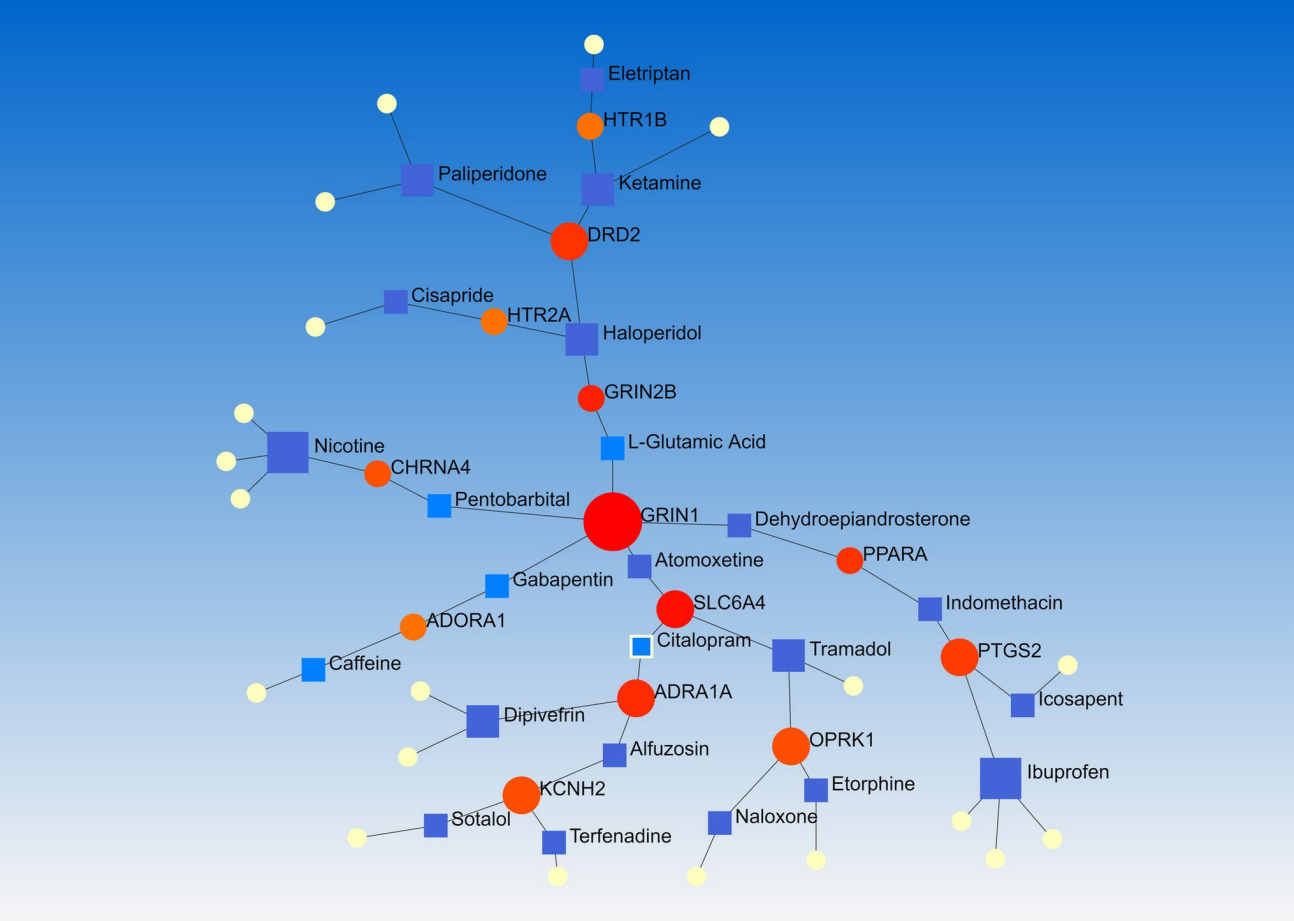
The PDIs of 55 genes in a Fruchterman–Reingold network showing the most potential drugs in association with the PGx of Opioid prescription in Spine pain managements

Analyzing 55 final genes by NetworkAnalyst (according to DrugBank ver. 5) adjusted for senior network, Fruchterman–Reingold model of PDIs showed important associated drugs. Drugs with the highest degree of betweenness are Ibuprofen and Nicotine with a degree of 4; Tramadol, Dipivefrin, Haloperidol, Ketamine, and Paliperidone with a degree of 3; and l-Glutamic Acid, Icosapent, Caffeine, Citalopram, Eletriptan, Atomoxetine, Pentobarbital, Indomethacin, Terfenadine, Naloxone, and Alfuzosin with a degree of 2. The other drugs can be found on Fig. 4.

### Protein–Chemical Interactions (PCIs)

**Fig. 5:**
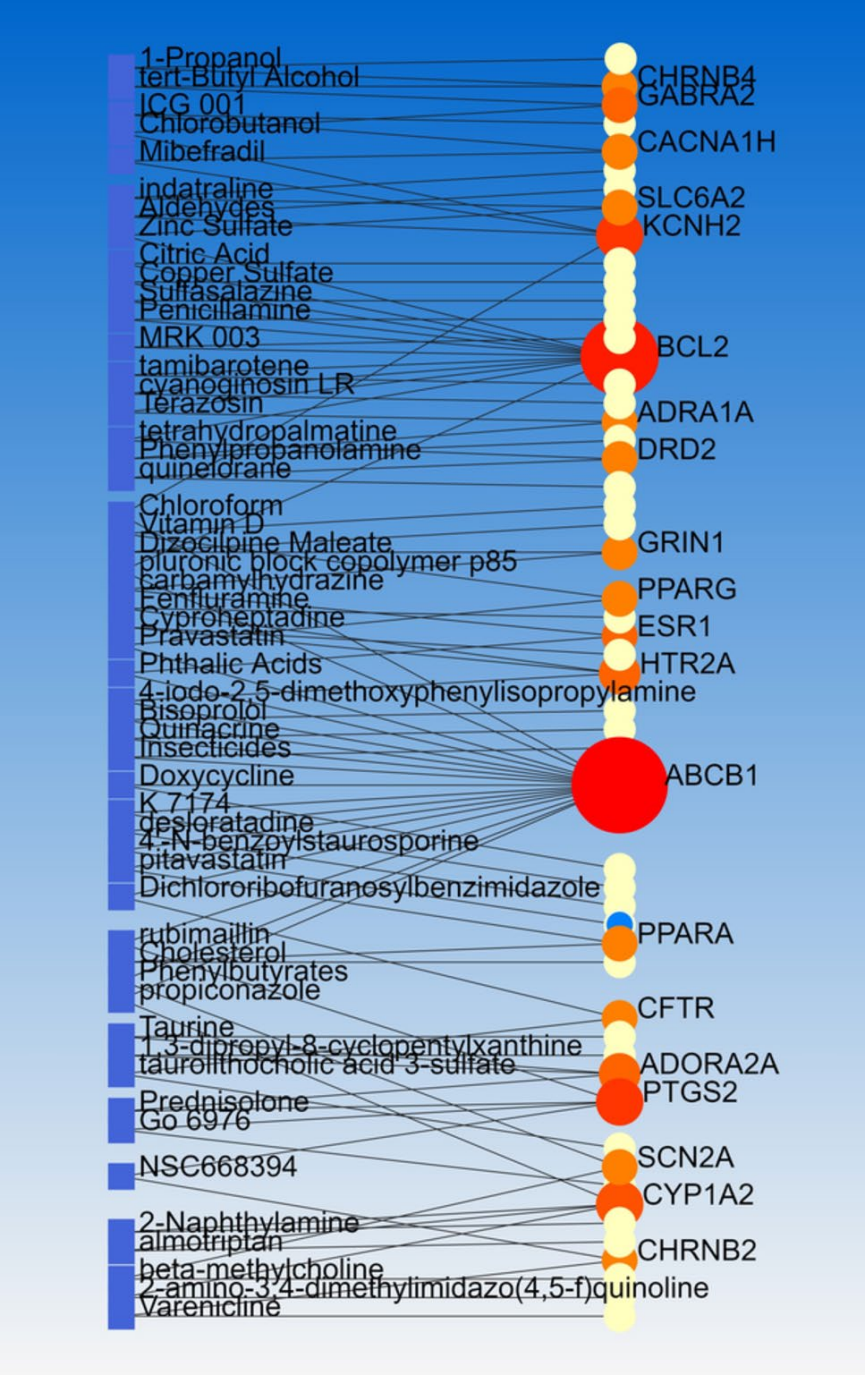
Linear Bi/Tripartite model of PCIs visualized by NetworkAnalyst

PCIs carried out by NetworkAnalyst based on data from the Comparative Toxicogenomics Database (CTD). Linear Bi/tripartite model adjusted for Steiner Forest Model which indicated at least one chemical for each of 55 target genes. Notably, some target genes had more than 2 interaction including ABCB1 (17 interactions), BCL2 (11 interactions); CYP1A2, KCNH2, and PTGS2 (4 interactions); and some of them had 2 interactions for example DRD2. Thus, ABCB1 was the most important target of suggested chemicals. Additionally, 10 chemicals were found with two interactions with the mentioned target genes including carbamylhydrazine, tetrahydropalmatine, 4-iodo-2, 5-dimethoxyphenylisopropylamine, 2-amino-3, 4-dimethylimidazo(4, 5-f)quinolone, Terazosin, beta-methylcholine, propiconazole, rubimaillin, quinelorane, and 1,3-dipropyl-8-cyclopentylxanthine (Fig. 5).

### Literature Review

**Fig. 6:**
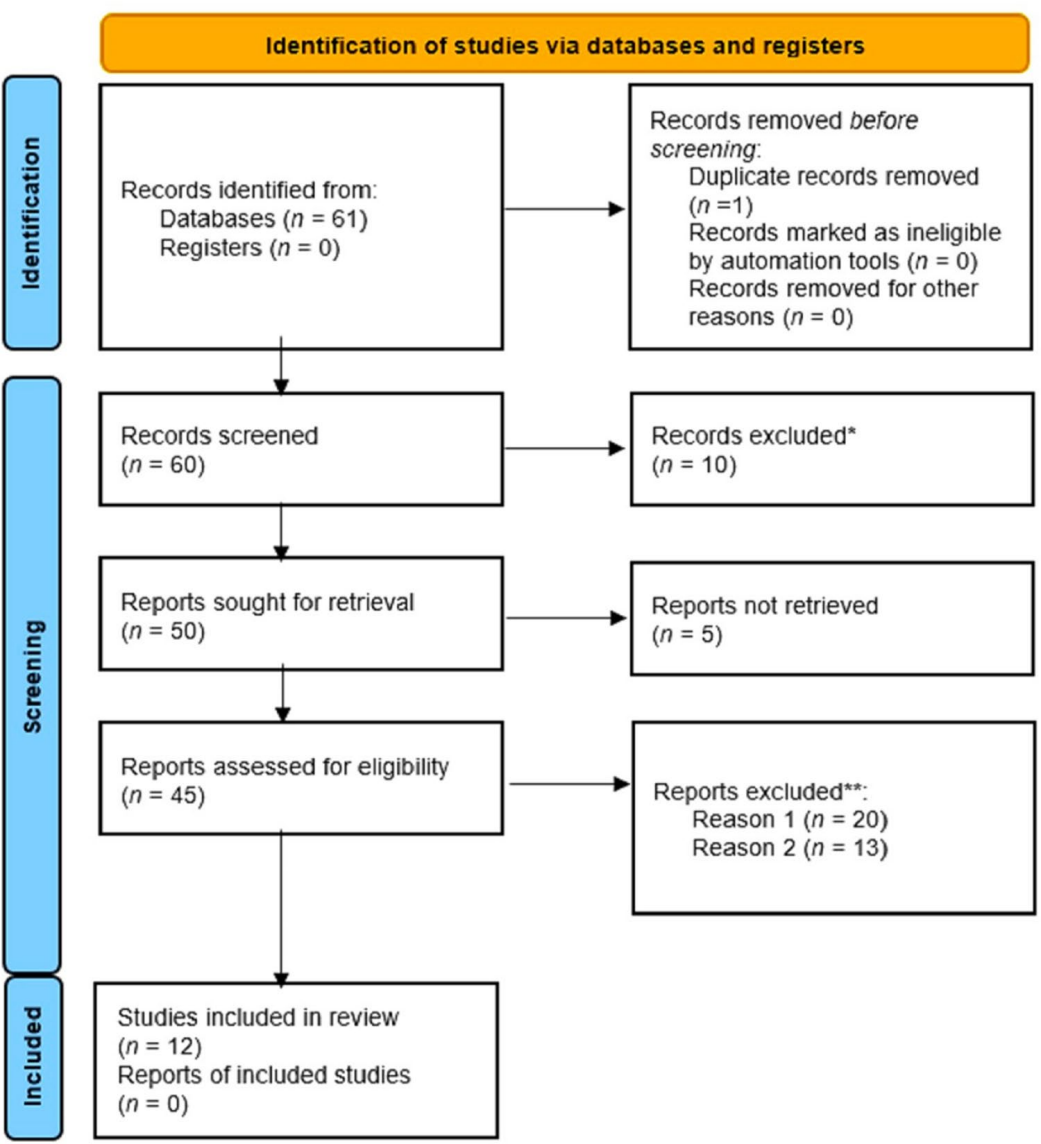
PRISMA workflow of reviewed studies. *These studies did not include the opioids prescription in surgical operations. **These publications were discarded for Reason 1: opioids in surgery were not their main focus; and Reason 2: they did not about orthopedic surgeries and opioid prescription

According to MeSH (Medical Subject Headings), we searched PubMed using the MeSH guidelines, and selected the titles and subheading of the papers for inclusion criteria (https://pubmed.ncbi.nlm.nih.gov/help/#using-mesh-database). Reviewing the PubMed database by focusing on the related terms including Pharmacogenomics, opioid, spine, and surgery revealed that there are 42 publications with the keywords of ‘Pharmacogenomics’ and ‘Opioids’ from 2011 to 2023; 16 papers with the keyword of pharmaocogenomics, opioid, and ‘surgery’ from 2008 to 2023 and 3 papers containing pharmacogenomics, opioid, and spine from 2014 to 2023; notably, 2 articles by Cottrill reported similar results. Following exact examination of related publications to this review, 12 papers were relevant and are examined in the discussion section. Figure 6 summarized a PRISMA workflow containing the details.

## Discussion

Technology advancements have mitigated many problems related to traditional open spine surgery related to large incisions, extended surgery times and excessive blood loss contributing to postoperative pain. Robotic-guided, navigation-guided, enhanced reality-assisted, minimally invasive and endoscopic spine surgery alternatives have been shown to decrease tissue disruption and operative times (Carrau et al. 2013; Snyder 2018; Galetta et al. 2019), potentially resulting in a shorter length of stay and an accelerated overall recovery (McClelland and Goldstein 2017).

In addition to these innovative surgical techniques, implementation of enhanced surgical recovery (ERAS) protocols has modernized the way spine surgery patients prepare for, undergo, and recover from surgery (Carr et al. 2019; Wang et al. 2019). Sufficient pain control can be obtained through the combination of preoperative education on pain management and improving intraoperative non-opioid pain-management strategies. Employing these pain management strategies in an integrated approach may allow to minimize the use of postoperative opioids significantly.

Moreover, by determining certain patient information, such as (I) preoperative opioid vulnerability according to an individual's genome, (II) surgery type, (III) age, (IV) BMI, (V) depression/anxiety detections, (VI) duration of hospital stay, and (VII) pain scores at hospital discharge, are capable of more accurately predicting opioid consumption patterns. Ideally, prescription methods may be tailored according to the requirements of an individual (Dufour et al. 2014; Gao et al. 2021), recognizing that the causes of the opioid crisis are multifaceted, with socioeconomical determinants of health playing an important part (Orosz and Yamout 2023).

Genetic study on opioid analgesic response has concentrated on selecting SNPs in candidate genes (encoding proteins) which are associated with identified opioid pharmacokinetics and pharmacodynamics and those related to alternate pain-related pathways (Lötsch and Geisslinger 2006; Kim et al. 2009). Abundant studies have confirmed differing analgesic responses related to candidate SNPs, remarkably increased postoperative morphine requirements among adults homozygous for μ opioid receptor (MOR) variants which is encoded by *OPRM1* A118G, but the findings do not carry across all patient populations (Chou et al. 2006a, b; Chou et al. 2006a, b; Coulbault et al. 2006; Janicki et al. 2006). Current candidate gene approaches give clear evidence of the polygenic nature of postoperative pain and opioid analgesic requirement, but continue to limit findings to known pain pathway elements (Coulbault et al. 2006; Mamie et al. 2013). A remarkable research by Chen et al. hypothesized that brain reward circuitry genes were genetic antecedents of pain sensitivity and critical diagnostic and also, they focused on pharmacogenomic treatment targets for chronic pain conditions. They introduced the concepts of brain reward cascade (BRC) and reward deficiency syndrome (RDS) both playing vital roles in pain management by opioids. A noticeable number of genes including OPRD1, OPRK1, OPRM1, BDNF, DRD genes (D1–5), MAO-A, COMT, PDYN, CYP2D6, CYP2B6, CYP2C19, CYP2C9, ABCB1, PENK, CNR1, UGT2B7, TTC12, ANKK1, NCAM1, ZCRB1, GABA(A), the metabotropic receptors mGluR6 and mGluR8, nuclear receptor NR4A2 and cryptochrome 1 (photolyase-like), Dat1, DBH, CAMKII; GnRH; NT-3 genes; G-protein alpha subunits; alpha2-adrenoceptor; interleukin-2; RGS-R7; Gbeta5, serotonin transporter, Ca^2+^/cAMP responsive element binding protein; P-glycoprotein, and CREB (Chen et al. 2009) have been implied in BRC and RDS.

Here we systematically reviewed 61 publications and found 12 relevant PubMed-indexed publications which are discussed in the following. By studying five tag SNPs including A118G, IVS2 + G691C, IVS3 + G5953A, IVS3 + A8449G, and TAA + A2109G, Hayashida et al. reported that analgesic necessities following major abdominal surgery are associated with both genotype and haplotype of OPRM1 gene polymorphisms (Hayashida et al. 2008). Fukuda et al. investigated the association of OPRM1 gene polymorphisms (118G and IVS3 + A8449G) with fentanyl susceptibility among patients experiencing painful cosmetic surgery. Their results offered insight into the role of OPRM1 3′ untranslated region (3′-UTR) polymorphisms, together with the A118G SNP, in fentanyl sensibility, and opened up new options for individualized pain management with fentanyl (Fukuda et al. 2009). Cook-Sather et al. conducted the first experimental GWAS in a pediatric day surgery cohort and found no relationships between rs795484 and rs1277441 at the TAOK3 gene with total morphine request in European Caucasian children (Cook-Sather et al. 2014). We investigated the TAOK3 pharmacogenomics associations in PharmGKB and then included this gene with 55 candidate genes from our analyses, but it showed no molecular connection with any members of candidate genes in a PPIs network by STRING-MODEL. Thus, we excluded TAOK3 from our list. Zhang et al. examined the impact of CYP3A5*3 polymorphism and the association between CYP3A5*3 and CYP3A4*1G variants on fentanyl analgesia following gynecological surgery among Chinese patients. They showed that, in Chinese women having gynecological surgery, CYP3A5*3 was not the primary genetic factor that contributed to inter-individual variation in the post-operative analgesic impact of fentanyl; however, a relationship between CYP3A5*3 and CYP3A4*1G variants can profoundly affect postoperative pain (Zhi-Xue et al. 2023). The influence of CYP3A5*3 polymorphism and interaction between CYP3A5*3 and CYP3A4*1G polymorphisms on post-operative fentanyl analgesia was highlighted in Chinese patients (Zhang et al. 2011). Senagore et al.'s study was designed to conduct the first evaluation of the effects of a PGx-guided analgesic option in an Enhanced Recovery Protocol (ERP) after major abdominal surgery (Zhao et al. 2022). They investigated the genes OPRM1, COMT, CYP2C9, CYP2C19, CYP1A2, CYP2D6, CYP3A4, CYP3A5, and ABCB1. According to their statistics, the groups' gender and procedure mixes were comparable, and over half of the PGx group experienced altered analgesia from the conventional ERP. Post-Op Day 1 (POD 1) (3.8 vs. 5.4) to POD 5 (3.0 vs. 4.5) showed substantially lower Overall Benefit of Analgesia Score (OBAS) ratings (*p* = 0.0.1) for the PGx group. In POD 1 to POD 5, the PGx group's analgesia was additionally better (*p* = 0.04). They came to the conclusion that 80% of patients had narcotic alterations of the ERP analgesic scheme and 56% of patients had NSAID selection based on pharmacogenetic advice utilizing the multi-gene profile analyzed. Compared to their normal ERP, these adjustments produced good analgesia with a 50% decrease in narcotic intake and a reduced risk of analgesic-related adverse reactions. These findings pointed to potential areas of improvement for ERP deriving from a patient-centered, lower-narcotic analgesic regimen that offers early and long-lasting pain relief with fewer adverse effects associated with opioid use (Senagore et al. 2017). Rocco et al. hypothesized that pharmacogenomic detection of individuals with varying opioid metabolism capability might allow for individualization of post-surgical opioid prescriptions. This study proposed an association between genetically regulated metabolism and opioid needs following surgery. Enhanced CYP2D6 enzymatic activity was linked to higher opioid intake, fewer unused opioids, and worse satisfaction with opioid prescriptions (Rocco et al. 2019). Li et al. identified 27 SNPs from 9 genes, including ABCB1, COMT, ARRB2, DRD2, MC1R, KCNJ6, OPRM1, OPRD1, and UGT2B7, that matched selection criteria and were evaluated with TAOK3. Morphine dose in African American (AA) children and ABCB1 rs1045642 (*p* = 0.02) and OPRM1 rs1799971 (*p* = 0.02); KCNJ6 rs2211843 and high pain in AA subjects (*p* = 0.01) and in congruent European Caucasian pain phenotypes (*p* = 0.01); and COMT rs740603 for high pain in European Caucasian individuals (*p* = 0.046). According to Li et al., TAOK3 (rs795484) is still a major contributor to high morphine dosage requirements in European Caucasian people (Li et al. 2019a, b).

Along these lines, Ettienne et al. (2019) importantly, conducted a retrospective cohort study of 113 patients undergoing buprenorphine-based OUD management in Northeast Washington, DC to find if clinical pharmacogenomics testing for CYP3A4 and CYP3A5 would influence treatment consequences. Clinical outcomes were based on presence of withdrawal symptoms, instances of unauthorized substances in urine drug tests (UDTs), and sublingual buprenorphine/naloxone (SBN) dose with standard-of-care (SOC) dosing versus pharmacogenomics (PGx)-based dosing. Their research suggested that patients with at least one copy of the CYP3A4*1B allele exhibit an accelerated rate of metabolism compared to the wild-type allele CYP3A4*1. These findings have translated in novel approaches in the treatment of pain and addiction by the same group (Ettienne et al. 2017). They suggested that pharmacogenomic testing as clinical decision support helped to individualize OUD management. They further suggested that collaboration by key stakeholders is essential to establishing pharmacogenetic testing as standard of care in OUD management.

Matic et al. examined the possible function of OPRM1 and COMT variants in acute, chronic, and experimental thermal pain following adult heart surgery. The subjects were randomly assigned to receive remifentanil or fentanyl throughout the procedure. They discovered that in adult cardiac surgery individuals, the COMT haplotype can partially account for the variation in early postoperative pain (Matic et al. 2020). Merchant et al. demonstrated that the association of CYP2D6 genotype can predict phenotypes with higher oxycodone requirements and adverse effects among children who underwent surgery. They genotyped *CYP2D6* alleles by the TaqMan allelic discrimination system in three allelic groups including full enzyme activity [*1 (N/A), *2 (rs16947), *2A (hCV32407252), *35 (rs769258)]; reduced enzyme activity [*9 (rs5030656), *10 (rs1065852), *17 (hCV2222771), *41 (hCV34816116)]; and none enzyme activity [*3 (hCV32407232), *4 (rs3892097), *5 (deleted), *6 (rs5030655), *7 (rs5030867), *8 (rs5030865), *11 (rs5030863), *14 (rs5030865), *15 (hCV32407245), *18 (hCV32407220), *19 (hCV32407233), *20 (hCV72649949), *40 (hCV32407240), *42 (hCV72649935), *44 (hCV32407228)]. Their results revealed that increasing parent oxycodone and oxymorphone might affect clinical outcomes in severe phenotypes, but oxymorphone might have a superior safety profile. They also discovered that CYP2D6 phenotype data accessible in electronic medical records (EMR) strongly affected oral opioid prescribing patterns (Merchant et al. 2022). Prashant and colleagues studied the association of pain perception and fentanyl consumption following major abdominal surgery with CGRP 4218T/C variant. They discovered that this variation influences postoperative pain perception and analgesic intake. Patients who carried the C/C genotype had greater postoperative fentanyl intake and pain ratings, according to the researchers (Prashant et al. 2023).

To the best of our knowledge based on PubMed database, publications related to the pharmacogenomics in spine pain managements are limited to two reports including Chidambaran et al. (2015) and Cottrill et al. (2021) Chidambaran et al. performed a prospective genotype-blinded research on 88 healthy adolescents (aged 11–18 years; 85% Caucasian, 67% female) who had spine fusion surgery for scoliosis. They were observed for 48 h following surgery to assess pain levels, analgesic adjuvant usage, morphine intake, and morphine-induced respiratory depression (MIRD). OPRM1 A118G variant genotyping revealed that 76% of patients were wild type (AA) and 24% were heterozygous/homozygous for the variation (AG/GG). Results showed that the risk of MIRD in patients with AA genotype was significantly higher (*p* = 0.030) and presence of G allele was also associated with higher pain scores (*p* = 0.045) (Chidambaran et al. 2015). Cottrill et al. studied genotypes of genes including *CYP2D6, CYP1A2, CYP2C9, CYP3A4, CYP3A5, CYP2B6, CYP2C19*, and *UGT2B7*. Haplotypes were then utilized to assess each patient’s relative capability to metabolize commonly used analgesic drugs. These drugs included both non-opioid (for example ibuprofen, diclofenac, mefenamic acid, nabumetone, indomethacin, piroxicam, meloxicam, tenoxicam, lornoxicam, etoricoxib, parecoxib, celecoxib, flurbiprofen, ketoprofen, fenoprofen, hydrocodone, naproxen, and aspirin) and opioid analgesics (including morphine, ethylmorphine, hydromorphone, codeine, dihydrocodeine, methadone, hydrocodone, oxycodone, oxymorphone, alfentanil, fentanyl, sufentanil, meperidine, ketobemidone, dextropropoxyphene, levacetylmethadol, loperamide, buprenorphine, dextromethorphan, tramadol, tapentadol, and tilidine). They constructed the first pharmacogenetic profiles of an outpatient spine cohort and gave preliminary proof that an outpatient analgesic regimen consisting of one medication since they are poor metabolizers. They discovered that almost half of the patients were given one analgesic because they had genetic variants that caused them to be poor metabolizers of the medication. Given the existing opioid epidemic, 13 of the 19 patients (68%) who were taking opioid drugs at the time of examination were excessively fast metabolizers of their prescription opioid analgesic (Cottrill et al. 2021). The current review is the first report with a pharmacogenomics-based strategy of analyzing all FDA-approved drugs (as of 9 November 2023) by targeting protein-coding genes and filtering the structural and regulatory pharmacological actionable variants. Our report, in comparison with the most relevant reports by Chidambaran et al. (2015). and Cottrill et al. (2021), included more genes (128 genes were filtered down to 55 genes), and a comprehensive variant list, and multiple in silico analyses including PPIs, GMIs, PDIs, and PCIs leading to several crucial findings which are highly recommended to be investigated in future studies of opioid prescribing in spine pain managements.

Based on the strong molecular evidences, in silico outcomes represented 55 candidate genes and 302 potential actionable variants. Additionally, results of signaling pathways proposed the Monoamine GPCRs as the most significant signaling pathway of opioid prescribing in spine pain managements, GMIs suggested hsa-miR-16-5p as a prospective miRNA candidate for detection and prognosis, PDIs revealed Ibuprofen and Nicotine, Tramadol, Dipivefrin, Haloperidol, Ketamine, and Paliperidone as the highly-impacted drugs on opioid prescription, and PCI analysis indicated some target genes with more than two interaction including *ABCB1, BCL2, CYP1A2, KCNH2, PTGS2*, and *DRD2*. Also, 10 chemicals were found by PCIs which need more functional validations.

Hou et al. revealed that miR-16 plays a key role in the post-transcriptional regulation of the OPRM1 gene. They stated that morphine decreases the expression of miR-16-5p in lymphocytes, which may be reversed by the antagonist naloxone (Hou et al. 2016a, b). Remarkably, miR-16-5p inhibits MOR expression by binding to a position in its 3′ UTR that is situated within 8699 and 8719 nucleotides from the stop codon (Hou et al. 2016a, b). According to Melo et al., miR-16-5p downregulation serves as a post-transcriptional process via which morphine increases MOR receptor concentrations through stabilizing its mRNA. (Melo et al. 2018). In another report by Chen et al., they suggested that by targeting RAB23 and inhibiting p38 MAPK activation in rats, miR-16 relieves chronic inflammatory pain (Chen et al. 2016). Following subcutaneous capsaicin injection, Kusuda et al. discovered that miR-16 expression was raised in the dorsal root ganglion (DRG) but reduced in the dorsal spinal cord (Kusuda et al. 2011). Furthermore, miR-16 was predicted to target BDNF. The *BDNF* gene was additionally a direct target of the miR-183 and miR-206, both of which were found to be downregulated in neuropathic pain (Lin et al. 2014; Sun et al. 2017) Additionally, their down-regulation was correlated with an increase in the expression of BDNF. In the spinal cord Chronic Compression Injury (CCI) model, it was also demonstrated that miR-183 was downregulated in neurons and microglial cells (Xie et al. 2017). Consequently, in microglia, miRNAs were connected to the BDNF signaling pathway (Dai et al. 2018). According to Li et al., in the spinal cord of CCI rats, interleukin-1β and tumor-necrosis factor-α expression were downregulated with suppression of miR-15a and miR-16. Using bioinformatics, they discovered that a potential target gene for miR-15a and miR-16 is G protein-coupled receptor kinase 2 (GRK2), is a crucial regulator of neuropathic pain and inflammation. According to their final results, the GRK2 gene is targeted by miR-15a/16 expression suppression, which reduces the development of neuropathic pain (Li et al. 2019a, b). Finally, Toyama et al. showed that miR-16-5p is a circulating blood biomarker which is commonly downregulated by both hydromorphone and oxycodone treatment with a *p*-value of 0.003 (Toyama et al. 2017). Altogether, miR-16-5p can be further studied for a candidate biomarker in post-operative opioid prescribing in spine pain management along with the genotyping of potential variants based on each individual’s genomic vulnerabilities.

## Summary

Variants in patients might affect both pharmacokinetic and pharmacodynamic characteristics of the drugs, which define drug metabolism and effectiveness. (Scott et al. 2019). Several drugs, for example opioids have these two factors involved due to drug modifications via the cytochrome P450 (CYP450) system in the liver changing the biological activity of the substance. Prior study has indicated that such variants are rare (Smith 2009), but their incidence in individuals with spine disorders is unclear. Knowing the incidence of these variants may assist patients in achieving better results since identifying inappropriate pharmaceutical analgesic regimens during clinical assessment may help maximize nonoperative treatment. This advancement, in turn, might reduce healthcare expenses while improving patient satisfaction by saving certain patients from the risks related to surgical intervention (Downs et al. 2019; Cottrill et al. 2021).

## Conclusion

The current review aimed to investigate the impact of pharmacogenomics in opioid prescribing of postoperative patients who underwent spine surgery to manage the pain severity and reduce the use of opioids. This goal leads to the personalized medicine strategies of opioid use according to the individual’s genotypes dictated to the potential candidate variants. The outcomes of this study indicated 56 highly-impacted genes. Additional in silico analyses showed novel findings in PPIs, GMIs, PDIs, and PCIs which need further functional and clinical validations.

## Data Availability

The data that support the findings of this study are available on request from the corresponding author.
